# Lumbar Plexus Palsy Caused by Massive Psoas Hematoma Related to Vertebral Compression Fracture in a Patient with Liver Cirrhosis

**DOI:** 10.3390/diagnostics13010115

**Published:** 2022-12-30

**Authors:** Seong Hwan Ahn, Dae Kyun Kim, Seok Won Kim

**Affiliations:** Department of Neurosurgery, College of Medicine, Chosun University, Gwangju 61453, Republic of Korea

**Keywords:** lumbar plexus, psoas muscle, liver cirrhosis

## Abstract

Osteoporotic vertebral compression fractures (VCFs) are common injuries in elderly patients and are usually stable because only the anterior column is involved. However, neurological deterioration may complicate osteoporotic VCFs, and most of them are related to canal invasion. Liver cirrhosis (LC) and its related complications have been identified as risk factors for an increased bleeding tendency, which, in turn, is associated with increased morbidity and mortality risks. We herein present a rare case of an osteoporotic VCF and a massive psoas hematoma that resulted in lumbar plexus palsy in a patient with LC after a stable-type spinal injury. To our knowledge, this is the first reported case of lumbar plexus palsy attributed to a liver-cirrhosis-related massive psoas hematoma and a stable VCF after minor trauma. This case highlights the potential risk of severe neurological deficits related to this type of common and seemingly trivial injury. The possible pathophysiological mechanisms are discussed and the relevant literature is reviewed.

After slipping from a chair, a 63-year-old woman was admitted to the emergency room (ER) with right groin pain, severe lower back pain (LBP), and an inability to walk due to motor weakness in the right lower limb. The patient presented with a medical history of diabetes mellitus and alcoholic LC, which she has had since she was 48 years of age. She was aware that she had osteoporosis, but no specific treatment was given for her osteoporosis.

Physical examination revealed significant tenderness in the lower back and right inguinal area. Neurological examination revealed complete functional deficits in all muscles of the right lumbar plexus (including both femoral and obturator nerves) with hypoesthesia of the anteromedial part of the right lower limb and decreased knee reflex. Laboratory blood tests revealed severe anemia and thrombocytopenia (Hb: 4.1 g/dL, HCT: 23.3%, a platelet count of 115,000 µL, a prothrombin time of 16.6 s (normal range: 9.4–12.5 s), and an activated partial thromboplastin time of 38.0 s (normal range: 28–44 s).

Considering the patient’s low hemoglobin level, an emergent computed tomography (CT) scan of the abdomen and lumbar spine was performed to exclude hemorrhages. Enhanced CT scans revealed a massive psoas hematoma, measuring 10 × 7 × 11 cm, extending from L2 to L5. Contrast dye leakage into the hematoma due to active bleeding from the injured segmental artery was detected (Figure 1). Magnetic resonance imaging (MRI) and CT scan of the lumbar spine revealed a stable osteoporotic VCF at L3 and an old compression fracture at L1, where vertebroplasty had already been performed (Figure 2). DEXA bone mineral densitometry was performed and showed severe osteoporosis (mean T-score of femur neck and spine: −3.6).

A vascular intervention specialist was consulted and urgent angiography was performed for transcatheter arterial embolization (TAE). The contrast medium was slightly extravasated from the distal fine branch of the third lumbar segmental artery, and TAE was performed at the suspected site of the leakage. Successful embolization of the bleeding vessel was performed using Gelfoam particles (absorbable porcine skin gelatin) (Figure 3). Percutaneous or surgical drainage was not considered due to the increased risk of bleeding complications and the expected potential for spontaneous reduction based on the TAE findings. The patient was treated conservatively with rest, vitamin K infusions, tranexamic acid, fresh frozen plasma, and packed RBC. During her hospital stay, her overall condition and liver function continued to improve.

After two months, despite the complete resolution of the hematoma, functional deficits in the lumbar plexus musculature persisted. The patient exhibited no clinical signs of neurological recovery. Electromyography (EMG) revealed complete denervation of the quadriceps femoris (vastus lateralis, rectus femoris, and vastus medialis) and the adductor longus.

Osteoporotic VCFs after minor trauma are common in the elderly and are usually treated successfully with minimal risk of complications [1]. Although rare, they can accompany severe acute complications that are associated with poor clinical outcomes. Although an intramuscular hematoma can occur after unstable injuries, such as fracture dislocations, or spontaneously in patients under anticoagulant therapy or in those with hemophilia, it is rare in patients with VCFs. However, LC is commonly associated with coagulopathies, including thrombocytopenia and hypoprothrombinemia, that can lead to easy bruising and an increased bleeding tendency [2,3,4]. LC-related comorbidities may be linked to impaired liver functions with decreased fibrinogen levels and increased fibrinolysis, damaged systemic vessel walls, and deficient platelet aggregation and activation of the clotting cascade [5]. Our patient was diagnosed with lumbar plexus palsy caused by a massive psoas hematoma related to an osteoporotic VCF after minor trauma. LC coupled with minor trauma additively increased the risk of lumbar segmental artery injury and intramuscular hematoma in our patient. The mechanism of compression injury of the lumbar plexus caused by a retroperitoneal hematoma has already been established [6]. The lumbar plexus is formed by the first four lumbar nerves (L1–L4) deep within the psoas, and in a psoas hematoma, the femoral and obturator nerves can be co-compressed, resulting in diffuse lumbar plexus injury, although this is an uncommon complication [7]. Previously published cases of complete lumbar plexus injury secondary to compression by a psoas hematoma were mostly spontaneous hematomas related to the bleeding tendency in patients with hemophilia, leukemia, and disseminated intravascular coagulation [8]. Iatrogenic lumbar plexus palsy has also been reported after lumbar plexus block for analgesia during hip surgery in patients treated with enoxaparin [9].

Careful suspicion is necessary for lumbar plexus palsy caused by a psoas hematoma due to the various developing symptoms and potential neurological deficits. Patients usually complain of groin pain, LBP, and progressive difficulty in walking [10]. Our patient also complained of groin pain, LBP, and motor weakness after her low-impact trauma. We initially suspected an osteoporotic burst fracture with canal invasion. Lumbar CT and MRI revealed a large psoas hematoma. Moreover, because of a significant drop in hemoglobin (4.1 g/dL), an abdominal CT scan to exclude acute gastrointestinal bleeding was performed. Typically, considerable difficulties in the detection of psoas muscle hematomas can lead to unnecessary examinations or a delay in diagnosis.

No treatment has been established for psoas muscle hematomas in patients with LC. The treatment options include conservative care, TAE, or surgical removal. Treatment of psoas muscle hematomas in patients with LC is challenging considering the likelihood of hemostatic dysfunction and poor general condition. In our patient, we chose management via TAE because of the high surgical risk related to the patient’s LC, the evidence of active bleeding, and the acute drop in hemoglobin level. TAE can be the treatment of choice for this type of injury. For patients with LC, an accurate diagnosis of lumbar plexus palsy is particularly important so that timely and adequate treatment can be provided.

In our case, the patient’s comorbidities, including liver cirrhosis, diabetes mellitus, and excessive alcohol use, possibly increased the risk of a giant hematoma after a trivial injury. Thus, in patients with multiple risk factors, great care should be taken regarding vertebral injury because segmental artery injury may cause a massive psoas hematoma followed by lumbar plexus palsy. Moreover, efforts such as pharmacologic therapy and those made regarding lifestyle are needed to prevent vertebral compression fractures in patients with osteoporosis.

Although rare in patients with LC, lumbar plexus palsy should be considered even if the fracture itself is stable, such as an osteoporotic VCF after minor trauma, because segmental artery injury may cause a massive psoas hematoma.

## Figures and Tables

**Figure 1:**
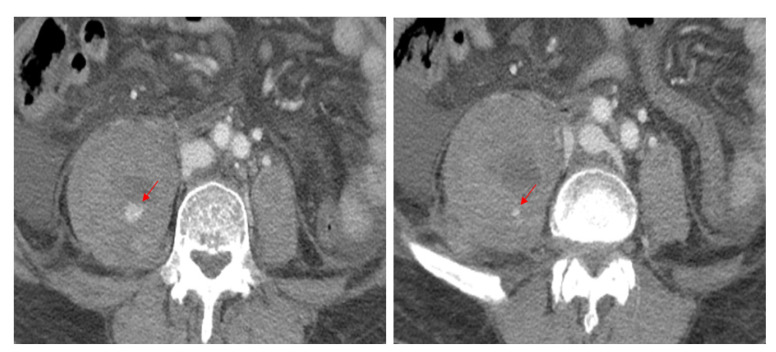
Contrast-enhanced CT scans reveal high attenuation, indicating active bleeding (arrows) from the right second and third lumbar arteries within the hematoma.

**Figure 2:**
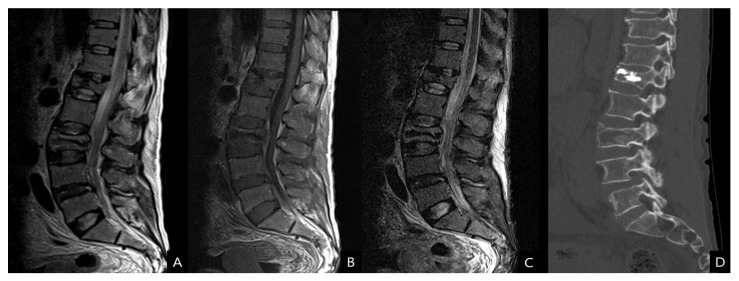
(**A**) T2-weighted, (**B**) T1-weighted, (**C**) short tau inversion recovery magnetic resonance images and also (**D**) computed tomography scan show a stable-type osteoporotic VCF at L3.

**Figure 3:**
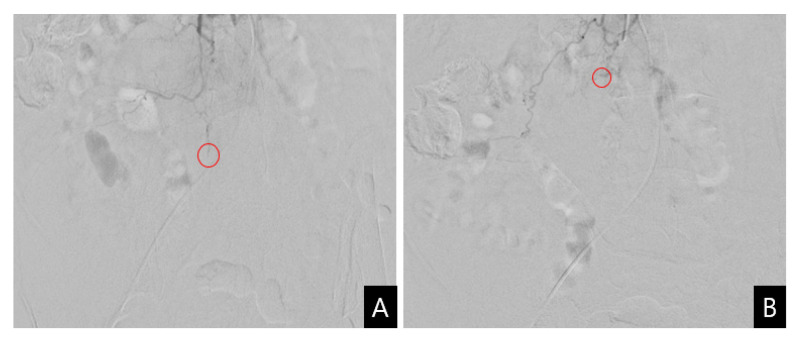
Transcatheter arterial embolization. (**A**) Selective angiography reveals the slightly extravasated contrast medium (circle) from the distal fine branch of the right third lumbar artery; (**B**) Embolization of the suspicious bleeding vessel is performed using Gelfoam particles (circle).

## Data Availability

Not applicable.

## References

[1] Longo U.G., Loppini M., Denaro L., Maffulli N., Denaro V. (2012). Conservative management of patients with an osteoporotic vertebral fracture: A review of the literature. J. Bone Jt. Surg. Br..

[2] Shin H.J., Ha S.W., Kim S.W. (2021). Delayed Onset Acute Subdural Hematoma after Burr Hole Drainage in a Patient with Chronic Subdural Hematoma and Liver Cirrhosis. Korean J. Neurotrauma.

[3] Di Bisceglie A.M., Richart J.M. (2006). Spontaneous retroperitoneal and rectus muscle hemorrhage as a potentially lethal complication of cirrhosis. Liver Int..

[4] Craxì A., Cammà C., Giunta M. (2000). Clinical aspects of bleeding complications in cirrhotic patients. Blood Coagul. Fibrinolysis.

[5] Grønbæk H., Johnsen S.P., Jepsen P., Gislum M., Vilstrup H., Tage-Jensen U., Sørensen H.T. (2008). Liver cirrhosis, other liver diseases, and risk of hospitalisation for intracerebral haemorrhage: A Danish population-based case-control study. BMC Gastroenterol..

[6] Goodfellow J., Fearn C.B., Matthews J.M. (1967). Iliacus haematoma. A common complication of haemophilia. J. Bone Jt. Surg. Br..

[7] Lee S.Y., Seok H., Kim H.J., Ahn J.Y., Kim S.H. (2020). Psoas Hematoma with a Large Pseudoaneurysm Causing Lumbar Plexopathy after Endoscopic Lumbar Decompression: A Case Report. J. Electrodiagn. Neuromuscul. Dis..

[8] Conesa X., Ares O., Seijas R. (2012). Massive psoas haematoma causing lumbar plexus palsy: A case report. J. Orthop. Surg..

[9] Klein S.M., D’Ercole F., Greengrass R.A., Warner D.S. (1997). Enoxaparin associated with psoas hematoma and lumbar plexopathy after lumbar plexus block. Anesthesiology.

[10] Yamashita S., Tanaka N., Nomura Y. (2012). Iliopsoas muscle hematoma secondary to alcoholic liver cirrhosis. Case Rep. Gastroenterol..

